# Fluorescent Radiosensitizing Gold Nanoparticles

**DOI:** 10.3390/ijms20184618

**Published:** 2019-09-18

**Authors:** Gloria Jiménez Sánchez, Pauline Maury, Lenka Stefancikova, Océane Campion, Gautier Laurent, Alicia Chateau, Farhan Bouraleh Hoch, Frédéric Boschetti, Franck Denat, Sophie Pinel, Jérôme Devy, Erika Porcel, Sandrine Lacombe, Rana Bazzi, Stéphane Roux

**Affiliations:** 1 Institut Univers, Temps-fréquence, Interfaces, Nanostructures, Atmosphère et environnement, Molécules (UTINAM), UMR 6213 CNRS-UBFC, Université Bourgogne Franche-Comté, 25030 Besançon CEDEX, France; gloria.jimenez.sanch@gmail.com (G.J.S.); gautier.laurent0@gmail.com (G.L.); youyin_08@hotmail.com (F.B.H.); rana.bazzi@univ-fcomte.fr (R.B.); 2 Institut des Sciences Moléculaires d’Orsay, UMR 8214 CNRS-UPS, Université Paris-Sud, 91405 Orsay CEDEX, France; pauline.maury@u-psud.fr (P.M.); llanayau@gmail.com (L.S.); erika.porcel@u-psud.fr (E.P.); sandrine.lacombe@u-psud.fr (S.L.); 3 Matrice Extracellulaire et Dynamique Cellulaire, MEDyC, UMR 7369 CNRS-URCA, Université de Reims Champagne-Ardenne, 51687 Reims CEDEX 2, France; Oceane.Campion@mrsolutions.com (O.C.); jerome.devy@univ-reims.fr (J.D.); 4 Centre de Recherche en Automatique de Nancy, Département Biologie, Signaux et Systèmes en Cancérologie et Neurosciences, UMR 7039 CNRS-UL, Université de Lorraine, 54505 Vandœuvre-lès-Nancy, France; alicia.chateau@univ-lorraine.fr (A.C.); sophie.pinel@univ-lorraine.fr (S.P.); 5 CheMatech, 21000 Dijon, France; fboschetti@chematech-mdt.com; 6 Institut de Chimie Moléculaire de l’Université de Bourgogne (ICMUB), UMR 6302 CNRS-UBFC, Université Bourgogne Franche-Comté, 21078 Dijon CEDEX, France; franck.denat@u-bourgogne.fr

**Keywords:** gold nanoparticles, radiosensitization, fluorescence imaging

## Abstract

Ultrasmall polyaminocarboxylate-coated gold nanoparticles (NPs), Au@DTDTPA and Au@TADOTAGA, that have been recently developed exhibit a promising potential for image-guided radiotherapy. In order to render the radiosensitizing effect of these gold nanoparticles even more efficient, the study of their localization in cells is required to better understand the relation between the radiosensitizing properties of the agents and their localization in cells and in tumors. To achieve this goal, post-functionalization of Au@DTDTPA nanoparticles by near-infrared (NIF) organic dyes (aminated derivative of cyanine 5, Cy5-NH_2_) was performed. The immobilization of organic Cy5-NH_2_ dyes onto the gold nanoparticles confers to these radiosensitizers fluorescence properties which can be exploited for monitoring their internalization in cancerous cells, for determining their localization in cells by fluorescence microscopy (a common and powerful imaging tool in biology), and for following up on their accumulation in tumors after intravenous injection.

## 1. Introduction

**Scheme 1:**
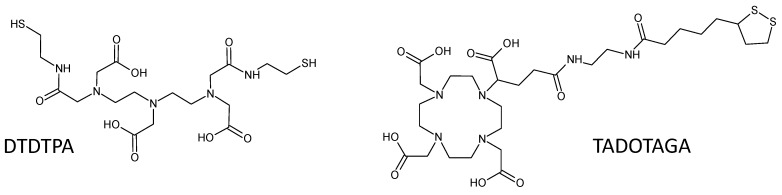
General structures of dithiolated derivative of diethylenetriaminepentaacetic acid (DTDTPA) and 1,4,7,10-tetraazacyclododecan-1-glutaric acid-4,7,10-triacetic acid functionalized by thioctic acid (TADOTAGA).

Among the numerous biomedical applications with gold nanoparticles (NPs) under consideration, the use as radiosensitizing agents for image-guided radiotherapy appears very promising, in particular in the case of ultrasmall gold nanoparticles (core diameter <3 nm) [1,2,3,4,5,6]. Radiotherapy, which is one of the three main treatments of cancer (applied alone or in combination with surgery and/or chemotherapy), consists of the eradication of cancerous cells using ionizing radiation (X- or γ-ray). Although it is commonly applied to treat a large range of cancers, radiotherapy is limited by a lack of selectivity that results from a behavior of normal and cancerous cells that is too similar when they are exposed to ionizing radiation. The chemical composition of normal and cancerous cells is too close to generate a difference in the X-ray absorption. In order to improve selectivity of the radiotherapy to be used, and therefore its efficacy, it has been proposed to exploit the local dose -enhancement induced by the interaction between nanoparticles containing elements with a high atomic number (Z) and the X-ray photons [1,2,3,4,5,6]. Nanoparticles are more suited than molecules because the biodistribution of nanoparticles is better controlled and each nanoparticle contains a larger amount of high-Z elements than molecules do (10–10^6^ vs. <10) [7,8,9,10,11,12,13,14]. As a result, the accumulation of the radiosensitizing nanoparticles in the tumor should favor the absorption of the ionizing radiation in a solid tumor. Such a preferential absorption will induce a cascade of physical and chemical reactions, which leads to a localized production of highly reactive species (radicals) [7,9]. The latter can generate lethal damage to cells. Such a strategy has been recently proven to be efficient for inhibiting tumor growth and sparing surrounding healthy tissues when irradiation was performed after the administration of gold-, platinum-, gadolinium-, or bismuth-based nanoparticles [4,5,6,10,11,15,16,17,18,19,20,21]. McMahon et al. demonstrated that ultrasmall gold nanoparticles are more efficient than large nanoparticles for enhancing the dose effect [7]. This conclusion is very interesting since nanoparticles with a hydrodynamic diameter of <10 nm can be removed from the body by renal clearance, which is a prerequisite to the in vivo application of non-biodegradable nanoparticles (such as gold nanoparticles) [22,23]. Besides the radiosensitizing effect, the ability to absorb X-ray photons can be exploited for monitoring the accumulation of the nanoparticles and therefore guiding the therapy [1,2,3]. The most opportune moment for inducing the irradiation can indeed be determined on the basis of data collected by X-ray imaging. However, X-ray imaging is probably not the most appropriate imaging modality for guiding radiotherapy owing to its low sensitivity and ionizing character. In order to overcome the limitations of X-ray imaging, gadolinium, indium, or technetium chelate-coated gold nanoparticles (Au@DTDTPA and Au@TADOTAGA) have been developed [24,25,26,27,28,29,30]. These nanoparticles are composed of an ultrasmall gold core (2 to 3 nm) encapsulated in a shell of linear (DTDTPA) or macrocyclic (TADOTAGA) polyaminocarboxylate ligands (Scheme 1).

**Scheme 2:**
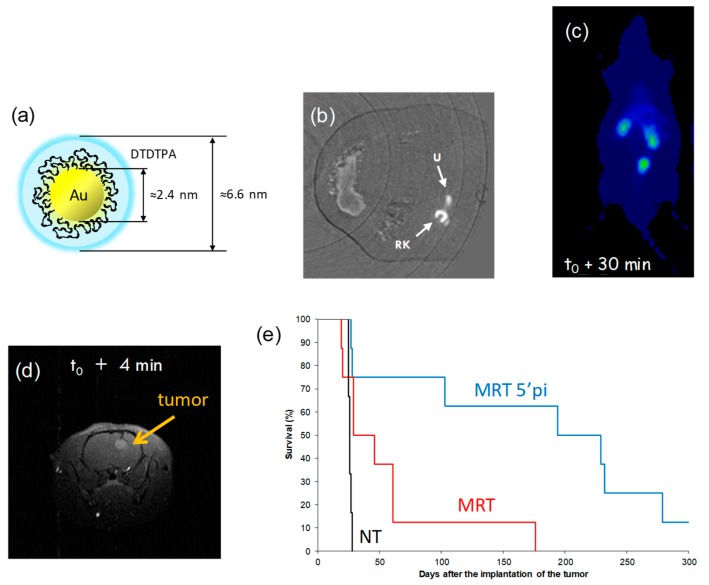
Illustration of Au@DTDTPA nanoparticles, properties, and applications. (**a**) Schematic representation of Au@DTDTPA nanoparticles. Behavior of Au@DTDTPA nanoparticles after intravenous injection into rats without a tumor (**b**,**c**) and with 9L gliosarcoma in the brain (**d**) as (**b**) contrast agent for X-ray imaging, (**c**) radiotracer for scintigraphy (after immobilization of ^99m^Tc ions in the organic DTDTPA shell), (**d**) positive contrast agent for MRI (after immobilization of gadolinium ions in the organic DTDTPA shell), and (**e**) efficient radiosensitizer for improving the survival of 9L gliosarcoma-bearing rats (tumor in the right hemisphere of the brain). NT, no treatment (no nanoparticle, no irradiation); MRT, only radiotherapy; MRT 5′pi, radiotherapy 5 min after intravenous injection of Au@DTDTPA(Gd) nanoparticles.

These ligands are dithiolated derivatives of diethylenetriaminepentaacetic acid (DTPA) and 1,4,7,10-tetraazacyclododecane-1,4,7,10-tetraacetic acid (DOTA) chelators that are well known for their ability to form highly stable complexes with gadolinium and indium ions [31]. As a result, Au@DTDTPA and Au@TADOTAGA nanoparticles behave as positive contrast agents for magnetic resonance imaging (MRI) and as radiotracers for nuclear imaging (planar scintigraphy and single-photon emission computed tomography (SPECT)) when they are labeled by gadolinium and technetium ions, respectively (Scheme 2) [25,26,27,28,29]. The data collected from SPECT (highly sensitive) and MRI (high spatial resolution) showed that these nanoparticles freely circulate after intravenous injection (i.e., no accumulation in healthy tissue) and are relatively quickly cleared renally and that a small fraction of them is temporary retained in the solid tumor [28,29]. On the basis of the images, a temporal window for the radiotherapeutic treatment was determined for an optimal exploitation of the radiosensitizing effect of the gold nanoparticles present in the solid tumor. When rats bearing a 9L gliosarcoma (9LGS, a brain tumor) in the right hemisphere of the brain are irradiated 5–10 min after intravenous injection of Au@DTDTPA(Gd) or Au@TADOTAGA(Gd), the life span of these diseased animals is increased by a factor 5 in comparison to non-treated animals and by a factor 2 in comparison to animals treated only by radiotherapy (Scheme 2) [28,29].

Although X-ray imaging, MRI, and SPECT are powerful imaging modalities which provide complementary meaningful information on the distribution of these gold nanoparticles in vivo, they are not suited for cell imaging. However, an improvement of the radiosensitizing efficiency of Au@DTDTPA nanoparticles requires a better comprehension of the impact of gold nanoparticles on cells when treated by radiation. This implies collecting information on the localization of the nanoparticles in the cells. This can be achieved by using fluorescence imaging. In this perspective, the functionalization of Au@DTDTPA nanoparticles by the aminated derivative of cyanine-5 (Cy5-NH_2_), which is a near-infrared (NIR) organic dye, is a crucial issue since the immobilization of fluorescent molecules onto gold nanoparticles will allow a visualization by fluorescence microscopy (a common and efficient technique for imaging cells) and also in vivo follow-up by fluorescence imaging (a powerful tool for preclinical studies) [32].

In this manuscript, we report on the modification of the radiosensitizing gold nanoparticles (Au@DTDTPA) with aminated NIR organic dyes (Cy5-NH_2_) and on their follow-up both in cells and in a living organism by fluorescence imaging.

## 2. Results and Discussion

### 2.1. Synthesis of Fluorescent Gold Nanoparticles

**Scheme 3:**
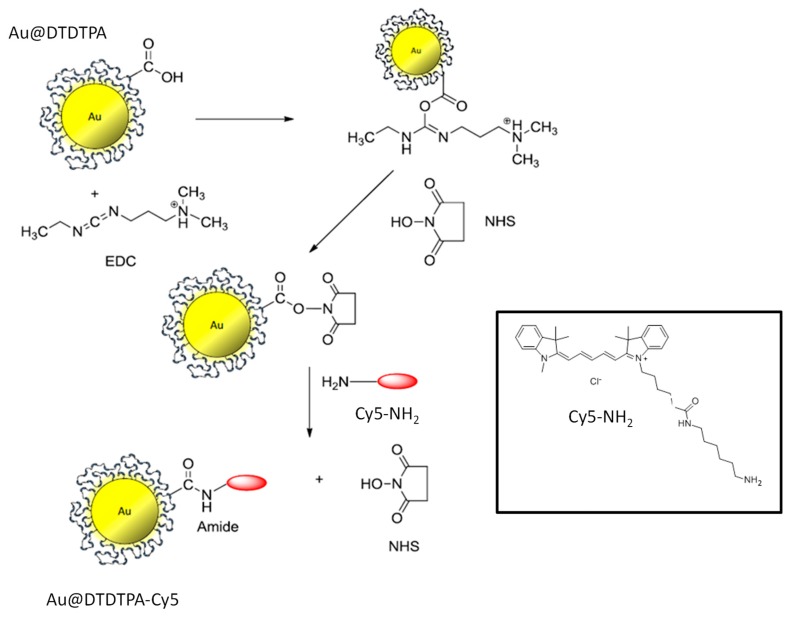
Functionalization of Au@DTDTPA with Cy5-NH_2_.

Since each DTDTPA contains three carboxylic acid groups, the latter can be used as a grafting site for the covalent immobilization of the aminated derivative of Cy5 (Cy5-NH_2_). The functionalization of Au@DTDTPA nanoparticles by Cy5-NH_2_ was therefore based on the formation of amide bonds, which results from the condensation between carboxylic acid and amine functions (Scheme 3).

**Figure 1:**
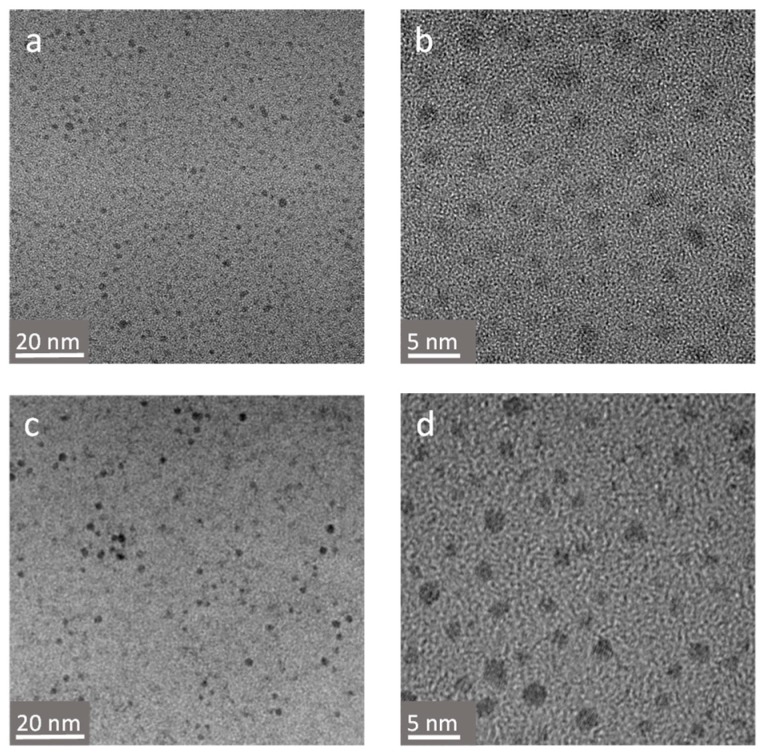
Transmission electron micrographs at different magnifications of (**a**,**b**) Au@DTDTPA and (**c**,**d**) Au@DTDTPA-Cy5 nanoparticles.

This reaction performed in aqueous media was promoted by *N*-(3-Dimethylaminopropyl)-*N*′-ethylcarbodiimide (EDC) and *N*-hydroxysuccinimide (NHS) [33]. After purification of the colloid by dialysis against an acid aqueous solution, transmission electron microscopy (TEM) experiments show that the reaction with Cy5-NH_2_ does not induce, as expected, any change in the morphology and core diameter of the Au@DTDTPA nanoparticles (Figure 1).

**Figure 2:**
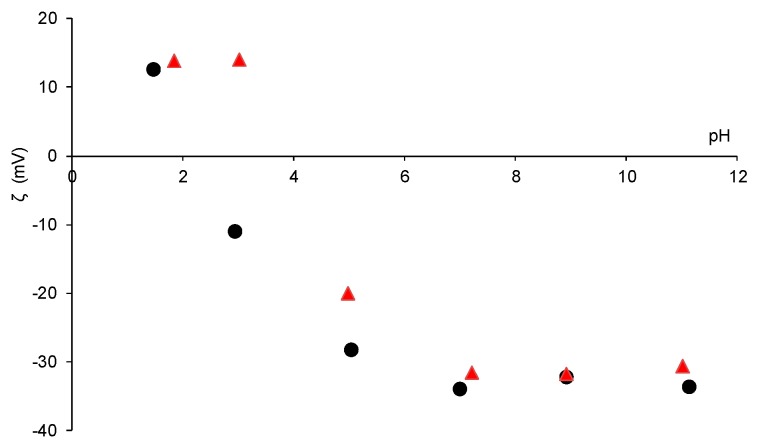
Zeta potential of Au@DTDTPA (black circles) and Au@DTDTPA-Cy5 (red triangles) as a function of pH.

Before and after the reaction, the core diameter was in the range of 2 to 3 nm. Despite the variation of pH imposed by the NHS ester chemistry (pH 5 for the activation step of COOH moieties and pH 7.5 for the grafting of Cy5-NH_2_), no agglomeration was observed. Such a behavior confirms the great colloidal stability of Au@DTDTPA nanoparticles for pH >3, which is mainly ensured by the electrostatic repulsion between charged nanoparticles. Au@DTDTPA nanoparticles exhibit a global positive charge for pH <3 and are negatively charged for pH >3 as reflected by the evolution of zeta potential as a function of pH (Figure 2). The pH dependent-charge of Au@DTDTPA nanoparticles can be explained by the nature of DTDTPA ligands anchored onto the gold core. DTDTPA is a polyaminocarboxylate derivative bearing three carboxylic acid (COOH/COO^−^) and three tertiary amine (R_1_R_2_R_3_NH^+^/R_1_R_2_R_3_N) groups. At low pH, the global positive charge of Au@DTDTPA nanoparticles stems from the predominance of protonated groups (COOH and R_1_R_2_R_3_NH^+^), whereas the negative charge of Au@DTDTPA nanoparticles observed for pH >3 is the consequence of the release of protons from the COOH and R_1_R_2_R_3_NH^+^ groups which yields COO^-^ and R_1_R_2_R_3_N. After reaction with Cy5-NH_2_, the evolution of zeta potential as a function of pH is similar to the one observed for non-functionalized Au@DTDTPA nanoparticles (Figure 2). However, the point of zero charge (pzc) is shifted to higher pH after the grafting reaction (from 2.3 to 4, Figure 2).

**Figure 3:**
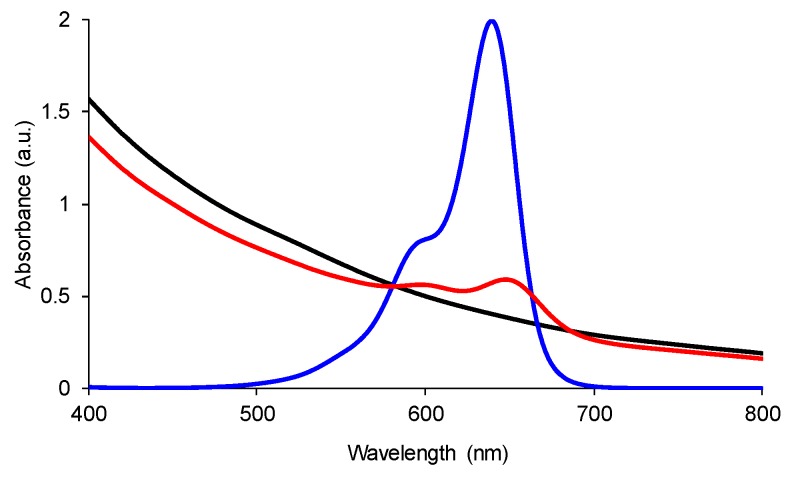
UV–visible spectra of aqueous suspensions containing Au@DTDTPA (black curve) and Au@DTDTPA-Cy5 (red curve) and of aqueous solution of Cy5-NH_2_ (blue curve).

**Figure 4:**
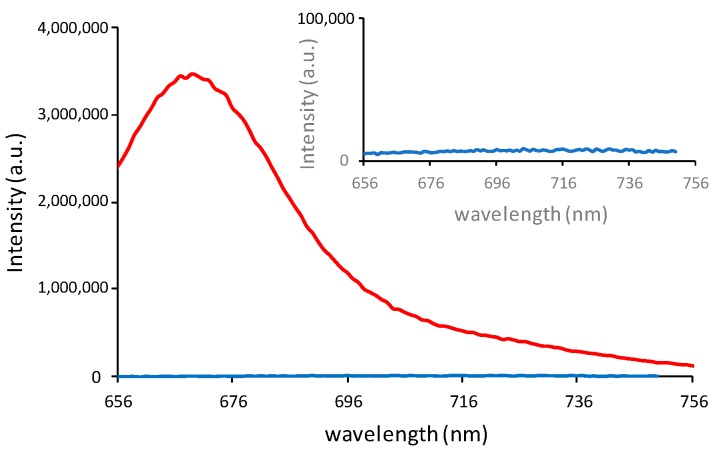
Emission spectra of aqueous suspensions containing Au@DTDTPA (blue curve) and Au@DTDTPA-Cy5 (red curve) (λ_exc_ = 646 nm). Inset: emission spectrum of the aqueous suspension containing Au@DTDTPA.

This shift can be attributed to the grafting of the NIR organic dyes which provide additional positive charges to the nanoparticles (Scheme 3). Despite the pzc shift, the great colloidal stability of Au@DTDTPA nanoparticles is preserved after the reaction with Cy5-NH_2_ in a large range around the physiological pH as reflected by the strongly negative values of zeta potential for pH >5. Another difference is revealed by UV-visible and luminescence spectra (Figure 3 and Figure 4). UV-visible spectra provide useful information on the size, the polydispersity, and the colloidal stability of gold nanoparticles. Gold nanoparticles with a core size larger than 5 nm are characterized by a strong absorption band assigned to the plasmon resonance phenomenon [34].

The position and the shape of the plasmon band depend on the size, the morphology, and the environment of the nanoparticles. However, this band is not observed for the Au@DTDTPA nanoparticles whose absorption spectra display a decrease of absorbance with a slight shoulder between 500 and 550 nm (Figure 3). The shape of the absorbance curve of Au@DTDTPA nanoparticles is characteristic of gold nanoparticles with core size <5 nm and confirms the data of TEM experiments [35,36,37]. The plasmon band is also not present in the absorption spectrum of Au@DTDTPA-Cy5, but two bands centered at 602 nm and 648 nm appear (Figure 3). In comparison to the UV-visible spectrum of Cy5, these bands can be attributed to the presence of Cy5 on the gold nanoparticles. It must be pointed out that a slight shift is observed when Cy5 is grafted onto the gold core (≈10 nm). As expected, the functionalization of Au@DTDTPA nanoparticles by Cy5-NH_2_ renders the gold colloid fluorescent. After excitation at 646 nm, the photoluminescence spectrum displays an emission band centered at 669 nm (658 nm for free Cy5-NH_2_), which is not visible on the spectrum of gold nanoparticles before the reaction (Figure 4).

The immobilization of Cy5 onto Au@DTDTPA nanoparticles is accompanied by a decrease in fluorescence lifetime (from 1.2 ns to 1.0 ns). When grafted to the gold nanoparticles, the fluorescence lifetime of Cy5 remains constant and different to the one of free Cy5 for at least 48 h. Such a difference indicates that there is no release of the organic dyes from Au@DTDTPA nanoparticles.

### 2.2. Internalization of the Au@DTDTPA-Cy5 Nanoparticles Monitored by Fluorescence Imaging

**Figure 5:**
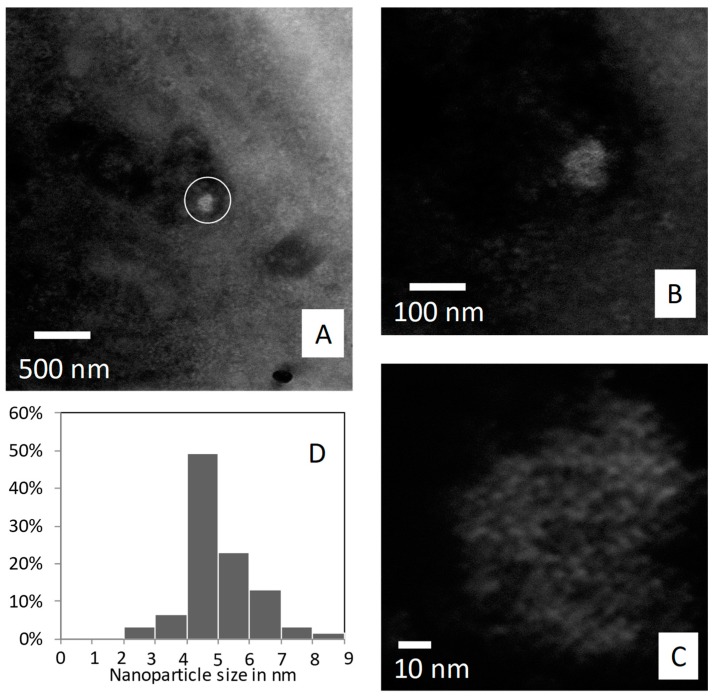
High-angle annular dark-field scanning transmission electron microscopy (HAADF-STEM) images. (**A**) Overall view of U87 MG cells containing Au@DTDTPA. The circle indicates a bright object containing atoms with a high atomic number that correspond to Au@DTDTPA. (**B**,**C**) High resolution images of the area in the circle of image A. (**D**) Size distribution of the bright object in the area in the circle of image A.

Owing to their small size (core diameter between 2 and 3 nm), the internalization of non-labeled Au@DTDTPA nanoparticles (i.e., without modification with organic dyes) is not easy to monitor with optical microscopy. High-angle annular dark-field scanning transmission electron microscopy (HAADF-STEM) was therefore used to characterize the intracellular localization of non-labeled Au@DTDTPA in U87 MG cells with high resolution (<10 nm). This technique uses the high atomic number of gold (Z = 79) to its advantage, compared with the elements from organic matter (H, C, N, O, P, S). Indeed, the images result from the electrons that cross the sample and are scattered at angles depending on the Z-numbers of the target atoms. Because the electrons are detected with an annular detector placed at variable height, the collection angle is set so that the contrast between elements of different Z is the maximum. To see the gold nanoparticles (Au@DTDTPA), a high-angle annular detector was used to obtain the signal of this high-Z element (white in the images). Several images were done in different cells and a signal corresponding to Au@DTDTPA was found in them, always in the cytoplasm. Images of U87 MG cells loaded with non-labeled Au@DTDTPA nanoparticles are shown in Figure 5. In the circle of Figure 5A, we can see a bright white zone in the cytoplasm of the cell, corresponding to the presence of the high-Z element. The zooms presented in Figure 5B,C show that this signal comes from small (<10 nm) high-Z particles located together in the cytoplasm. Their size determined from the HAADF-STEM images is slightly larger than the size of Au@DTDTPA nanoparticles measured from the TEM ones (about 5 nm in diameter vs. 2 or 3 nm) (Figure 5D). The difference (which remains moderate) can be explained by the lack of clarity at high magnification in HAADF-STEM images; this renders the measurements less accurate.

**Figure 6:**
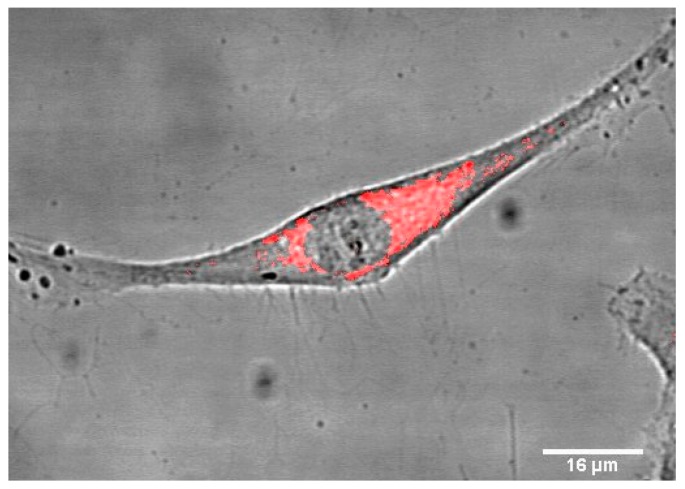
Confocal microscopy image of a glioblastoma cell (U87 MG) after 6 h of incubation with Au@DTDTPA-Cy5 nanoparticles (red), [Au]_incubation_ = 5 × 10^−4^ mol. L^−1^.

The fluorescence of the gold nanoparticles conferred by the grafting of the organic dyes allows monitoring their internalization in cells using less sophisticated means. After the incubation of glioblastoma cells (U87 MG) with Au@DTDTPA-Cy5, the superimposition of optical transmission and fluorescence images clearly reveals the presence of nanoparticles within the cytoplasm (Figure 6).

**Figure 7:**
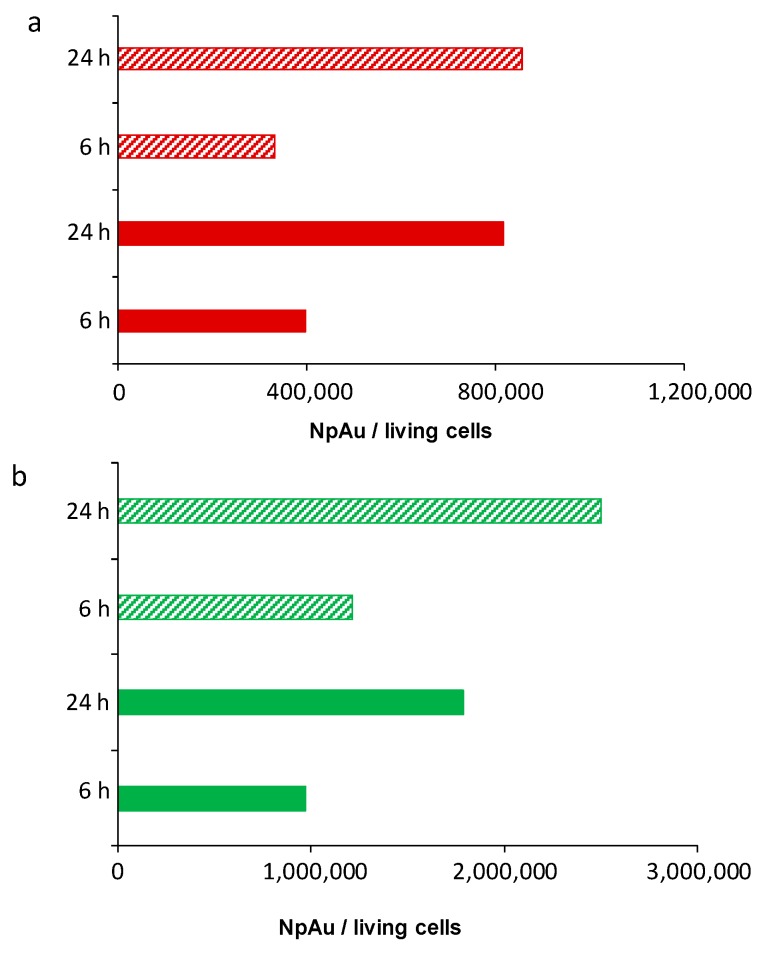
Inductively coupled plasma-optical emission spectrometry (ICP-OES) analyses of (**a**) HeLa and (**b**) U87 MG cells incubated with Au@DTDTPA (filled bars) and Au@DTDTPA-Cy5 (dashed bars) nanoparticles for 6 and 24 h.

Although these gold nanoparticles exhibit a very reduced size, no fluorescence is detected in the nucleus. The presence of gold nanoparticles was confirmed by inductively coupled plasma-optical emission spectrometry (ICP-OES) analysis (Figure 7). Whatever the cells, the internalization increases with the incubation time. However, the amount of internalized gold nanoparticles and the impact of the functionalization of the gold nanoparticles by Cy5-NH_2_ depend on the nature of the cells. The amount of gold nanoparticles and the impact of the functionalization on the internalization are lower in the case of HeLa cells than in the case of U87 MG cells. Even in the latter case, the impact of the functionalization on the internalization remains relatively low (<30%).

**Figure 8:**
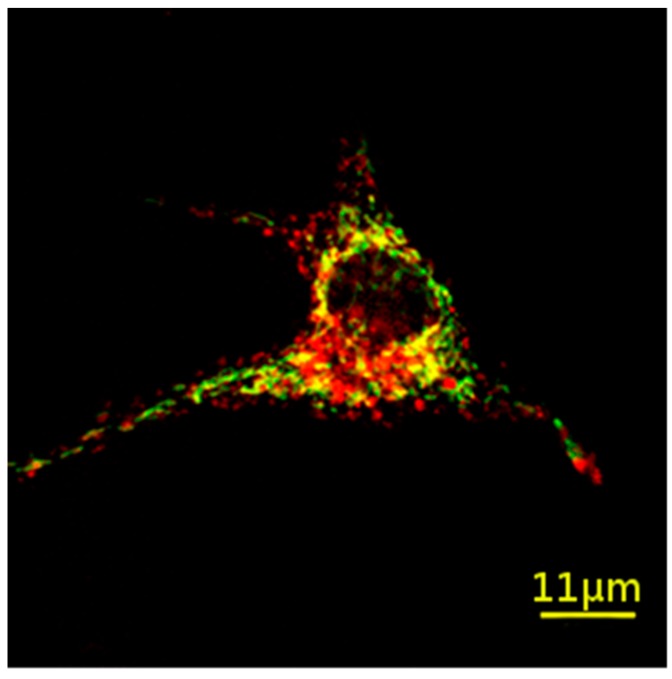
Fluorescence image obtained by confocal microscopy of U87 MG cell loaded with Au@DTDTPA-Cy5 nanoparticles (red) in the presence of MitoTracker Green (green). Yellow reflects the colocalization of fluorescent gold nanoparticles and mitochondria.

To better localize the NP action sites within the cell, colocalization studies of NPs and organelles were performed. In particular, the colocalization with mitochondria was measured using a fluorescent tracker (MitoTracker Green) having no spectral overlap with Cy5-labeled gold nanoparticles. Figure 8 shows a cell containing trackers (green) and Au@DTDTPA-Cy5 nanoparticles (red) after 6 h of incubation with the fluorescent gold nanoparticles.

In addition to green and red zones, large yellow zones that correspond to regions where red and green are both present can be observed (Figure 8). The apparition of yellow-colored zones reflects therefore the colocalization of gold nanoparticles and mitochondria. The presence of radiosensitizing nanoparticles in the vicinity of the mitochondria is an unexpected but very interesting result that may explain the ability of these ultrasmall gold nanoparticles to improve the efficiency of radiotherapy.

**Figure 9:**
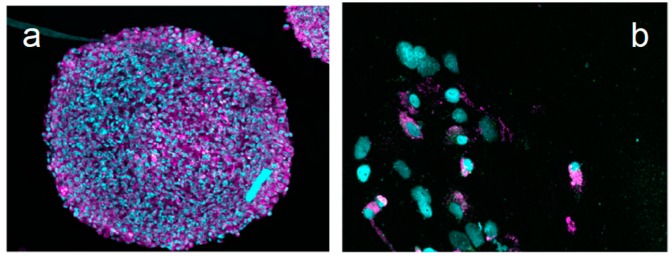
(**a**) Fluorescence imaging of a U87 MG cells spheroid section after 24 h exposure with Au@DTDTPA-Cy5 nanoparticles. (**b**) Fluorescence imaging of U87 MG single cells obtained after spheroid dissociation. Pink fluorescent signal corresponding to Cy5 was found in the cytoplasm, while cell nuclei were labeled with Hoechst 33342 (blue fluorescent signal).

Fluorescence imaging is not restricted to the observation of cells. This imaging modality is also a powerful preclinical tool since it can be applied for monitoring the diffusion of fluorescent nanoparticles in vitro in 3D cell culture and their biodistribution in animal models after intravenous injection [38,39]. Confocal microscopic images of U87 MG cells spheroid sections confirmed that Au@DTDTPA-Cy5 nanoparticles are able to reach the center of 800 µm diameter spheroids, highlighting the good diffusion abilities for these nanoparticles. In parallel, when the spheroids were dissociated to provide a single-cell suspension, a Cy5 fluorescence signal was observed in the cytoplasm of U87 MG cells, thus confirming the cellular uptake of Au@DTDTPA-Cy5 nanoparticles (Figure 9).

### 2.3. In Vivo Fluorescence Imaging

**Figure 10:**
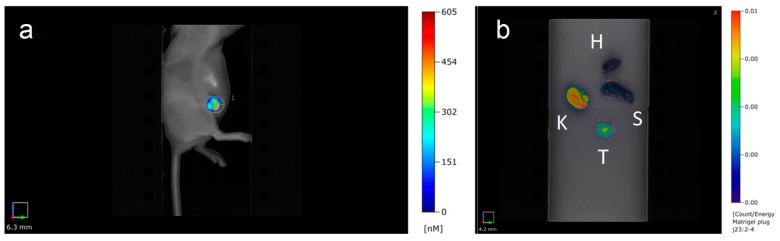
(**a**) Fluorescence imaging of a tumor-bearing mouse laid on its left flank after intravenous injection of Au@DTDTPA-Cy5 nanoparticles. (**b**) Fluorescence imaging of ex vivo organs (heart (H), kidney (K), spleen (S), and tumor (T)) after intravenous injection of Au@DTDTPA-Cy5 nanoparticles.

Furthermore, the post-functionalization of Au@DTDTPA by aminated Cy-5 NIR dyes open the door to their in vivo follow-up by fluorescence imaging. The image acquired 30 min after intravenous injection of the fluorescent Au@DTDTPA-Cy5 nanoparticles clearly shows a preferential accumulation in the tumor which appears highly fluorescent in comparison to the rest of the body (Figure 10). Ex vivo organ imaging shows an accumulation of Au@DTDTPA-Cy5 nanoparticles in kidneys and in the tumor, whereas no signal was detected in the heart or spleen. This preliminary in vivo fluorescence imaging study confirms, despite the surface modification, the safe behavior of these gold nanoparticles which was previously revealed by MRI and SPECT [27,28]. These nanoparticles are indeed characterized by a preferential accumulation in the tumor and also by renal clearance.

## 3. Materials and Methods

### 3.1. Au@DTDTPA Synthesis

The synthesis, based on the Brust method [40], consists of reducing a gold salt (HAuCl_4_·3H_2_O) with NaBH_4_ in the presence of thiols (stabilizers) that, by adsorption on growing particles, ensures control of the size and the colloidal stability. In this case, the chelator consists of a dithiolated derivative of diethylenetriaminepentaacetic acid (DTPA), named DTDTPA. The synthesis and characterization of the DTDTPA ligand has been described earlier [24,25].

Tetrachloroauric acid trihydrate (HAuCl_4_,3H_2_O), sodium borohydride (NaBH_4_), acetic acid (CH_3_COOH), hydrochloric acid, sodium hydroxide (NaOH), methanol, and other organic solvents (reagent grade) were purchased from Aldrich (Sigma-Aldrich Chemie S.a.r.l., Saint-Quentin Fallavier, France).

For a typical preparation of gold nanoparticles, HAuCl_4_·3H_2_O (200 mg, 51 × 10^−5^ mol) was placed in a 250 mL round-bottom flask and dissolved with methanol (60 mL). In another flask, DTDTPA (256 mg, 50 × 10^−5^ mol), water (40 mL), and acetic acid (2 mL) were mixed. This solution containing DTDTPA was added to the gold salt solution under stirring. The mixture turned from yellow to orange. NaBH_4_ (195 mg, 515 × 10^−5^ mole) dissolved in water (13.2 mL) was added to the gold–DTDTPA solution under stirring at room temperature. At the beginning of the NaBH_4_ addition, the solution first became dark brown then a black flocculate appeared. The vigorous stirring was maintained for 1 h before adding aqueous hydrochloric acid solution (2 mL, 1 M). After the partial removal of the solvent under reduced pressure, the precipitate was retained on the polymer membrane and washed thoroughly and successively with 0.1 M hydrochloric acid, water, and acetone. The resulting black powder was dried (up to 200 mg of dry powder of Au@DTDTPA) and dispersed in aqueous solution of sodium hydroxide (NaOH 0.01 M) to have a final concentration of 50 mM in gold.

### 3.2. Functionalization of Au@DTDTPA by NIR Organic Dye Cyanine-5-Amine (Cy5-NH_2_)

The preparation of the fluorescent-labelled nanoparticles (Au@DTDTPA-Cy5) was achieved by grafting near-infrared (NIR) organic dye (cyanine 5 derivative) onto the organic shell (DTDTPA) of the gold nanoparticles. Since the DTDTPA shell is rich in -COOH groups, aminated Cy5 (Cy5-NH_2_) was chosen for the functionalization of the gold nanoparticles. The condensation between -COOH groups of Au@DTDTPA nanoparticles and -NH_2_ of Cy5-NH_2_, which is promoted by EDC and NHS, yields amide function.

A solution of Au@DTDTPA (9 mL, 50 mM in gold) was adjusted to pH 5. For the activation of the carboxylic groups, EDC (397 mg) and NHS (477 mg) in deionized water (6.48 mL) were added to the colloid under stirring at room temperature. The agitation was maintained for 90 min. Afterward, the pH of the solution was adjusted to pH 7.5 and Cy5-NH_2_ (4 mg) was added to the aqueous suspension of Au@DTDTPA nanoparticles. The solution was stirred for 15 min at room temperature and for 12 h at 4 °C.

After the reaction with Cy5-NH_2_, the nanoparticles were purified by dialysis against acidic medium (pH 5, molecular weight cut-off (MWCO): 6 kDa). The dialysis bath was changed four times (6, 20, 26, and 40 h after the immersion of the dialysis tube in the acid aqueous solution) until it became colorless. After the purification by dialysis, the gold nanoparticles were concentrated by centrifugation using centrifugal concentrators (Vivaspin^®^, MWCO: 10 kDa) until a gold concentration of 50 mM.

### 3.3. Transmission Electron Microscopy

The size of the gold core was obtained from transmission electron microscopy (TEM) performed with a JEOL 2010 FEG microscope at 200 kV (INSA, Lyon, France) and a JEOL JEM 2100F microscope at 200 kV (ICB, Dijon, France). Drops of colloidal solutions were deposited on dedicated TEM carbon grids and observed after natural drying at room temperature. The treatment of the images and the determination of the size of the gold cores were achieved using Gatan DigitalMicrograph^TM^ software (3.10.01 for Gatan Microscopy Suite 1.5.1, Gatan, Pleasanton, CA, USA).

### 3.4. Measurements of ζ-Potential

The ζ-potential of the Au@DTDTPA and the Au@DTDTPA-Cy5 nanoparticles was directly determined using a Nanosizer ZS equipped with a He-Ne (633 nm) laser from Malvern Instruments at the Qualio Laboratory (Besançon, France). The colloids were diluted to obtain a concentration of 1 mM in gold (0.2 g Au/L), containing 0.01 M in NaCl and adjusted to the desired pH by the addition of NaOH or HCl 1 M.

### 3.5. Fluorescence Spectrometry

Fluorescence experiments were performed with a Fluorolog FL3-22 spectrometer (HORIBA Jobin Yvon, Kyoto, Japan) using Instrument Control CT software (2.2.13, Keysight Technologies, Santa Rosa, CA, USA), connected with the unit Spectra CQ. The wavelength of excitation was fixed at 646 nm and the scan was performed from 656 to 800 nm. The solutions were previously diluted to obtain a final concentration of 0.2 g Au/L and were analyzed in a standard quartz cuvette.

### 3.6. UV-Visible Spectrophotometry 

UV-visible absorption spectra were recorded at room temperature using a SPECORD 210 (Analytic Jena, Jena, Germany) and WinASPECT software (2.2.0.0, Analytic Jena, Jena, Germany). The samples were previously diluted to a final concentration of 0.2 g Au/L and were analyzed in a standard quartz cuvette. The scan was performed from 400 to 800 nm.

### 3.7. Inductively Coupled Plasma-Optical Emission Spectrometry (ICP-OES) 

The samples were mineralized in ultrapure aqua regia to a final concentration of at least 20 µg/L in gold. An ICP-OES (710 ES Varian/Agilent, Santa Clara, CA, USA) with axial torch with a concentric nebulizer and cyclonic spray chamber was used. The parameters fixed during measurement were as follows: power of 1.2 kW with argon auxiliary of 1.5 L/min and nebulizer pressure of 200 kPa. The emission lines used to measure the gold concentration were 267.594 nm, 242.794 nm, and 208.207 nm. The efficacy of the atomization is about 60% for gold. An ionizing buffer was employed for the measurements. The limit of detection of this technique is 20 µg/L.

### 3.8. Centrifugation

A Fisher Bioblock Scientific 2-16P centrifuge (Ilkirch, France) with a rotor 1251 was employed for concentrating gold nanoparticles suspension after purification (dialysis). The colloids were placed in the upper compartment of Vivaspin^®^ flasks equipped with a membrane (MWCO: 5 or 10 kDa). The centrifugations were performed at 238× *g* (1500 rpm).

### 3.9. Cell Culture

The HeLa cells (derived from cervical adenocarcinoma) and U87 MG cells (derived from glioblastoma) were purchased from ATCC France Office (Molsheim, France). They were cultivated in Dulbecco’s Modified Eagle Medium (DMEM) (Life Technologies, Carlsbad, CA, USA) supplemented with 10% heat-inactivated fetal bovine serum (Sigma Aldrich, Saint-Louis, MO, USA), 100 U/mL penicillin (Sigma Aldrich, Saint-Louis, MO, USA), 100 μg/mL streptomycin (Sigma Aldrich, Saint-Louis, MO, USA), and 1% nonessential amino acids (Life Technologies, Carlsbad, CA, USA). Cells were maintained in a 5% CO_2_ incubator (Heracell^®^, ThermoFischer, Langenselbold, Germany) at 37 °C.

To favor the formation of spheroids, U87 MG single cells were seeded at 4 × 10^4^ cells/mL in T75 culture dishes, with a hydrophobic poly(2-hydroxyethyl methacrylate) coating that prevents cell adhesion onto the bottom of the flask. Four days after seeding, the spheroids were transferred into a spinner (Dutscher, Brumath, France) and kept in culture for growing for at least ten days after seeding.

At the time of the experiments, U87 MG cells spheroids were incubated with 5 × 10^−3^ M Au@DTDTPA-Cy5 nanoparticles in 6-well plates for 24 h. The spheroids were rinsed twice with Hank’s Balanced Salt Solution (HBSS) after exposure to nanoparticles. Then, half of the spheroids were embedded into Tissue-Tek^®^ O.C.T. compound (Sakura^®^ Finetek, Staufen im Breisgau, Germany) and frozen in isopentane/ liquid nitrogen to prevent ice crystal formation. Fifty-micrometer cryostat sections were prepared, then fixed with formaldehyde 3.7%.

For the other half of the spheroids, enzymatic plus mechanical dissociation was performed using trypsin-EDTA 0.05% and a 21-gauge syringe needle to obtain a single-cell suspension without aggregates. Isolated cells were plated onto glass slides using cytocentrifugation. All slides (with spheroid sections or isolated cells) were then stained with Hoechst 33342, before observation with a confocal microscope.

### 3.10. High-Angle Annular Dark-Field Scanning Transmission Electron Microscopy (HAADF-STEM)

U87 cells were plated on glass coverslips. While attached, they were incubated with 1 mmol/L of Au@DTDTPA for 1 h. The cells were then rinsed with PBS 1× and fixed in the mixture of 2.5 % glutaraldehyde and 4% paraformaldehyde in PBS 1×. After rinsing the cells with PBS 1× and distilled H_2_O, they were dehydrated using ethanol in gradient concentrations until 100%. Next, the samples were embedded in resin step by step using a mixture of ethanol with increasing concentrations of Epon resin until 100%. After resin polymerization at 65 °C, the samples were cut using an ultramicrotome into 150 nm thick slices deposited on copper grids. The observation was performed on the microscopy platform IBiSA at the Institut Curie, Orsay, France with a Jeol 2200FS FEG electron microscope operating at 200 kV, using the 1nm probe and a camera length of 6 cm. ImageJ software (1.52a, National Institutes of Health, USA) (https://imagej.nih.gov/ij/, last accessed on: 23 April 2018) enabled a statistical analysis of internalized particles.

### 3.11. Confocal Microscopy

Confocal microscopy experiments were performed with a LEICA SP5 confocal system located at the Centre de Photonique Bio-Medical (CPBM), University Paris-Sud (Orsay, France) in a controlled chamber. The samples were kept at 37 °C and regulated in CO_2_. The U87 MG cells were incubated with 0.5 mM of Au@DTDTPA-Cy5 for 6 h. Cyanine 5 was excited at 633 nm and the fluorescence emission was detected in the 650–750 nm range. The images were processed with the freely available ImageJ software.

For colocalization studies, the U87 MG cells were incubated for 12 h with 200 nM MitoTracker Green (Invitrogen – Thermofisher Scientific, Waltham, MA USA). The trackers were washed out with PBS 1× before incubation for 6 h with 1 mM of Au@DTDTPA-Cy5. Organic dye was excited at 633 nm and the fluorescence emission was detected in the 650–750 nm range. The MitoTracker Green was excited at 488 nm and the fluorescence emission was detected in the 505–600 nm range. Images were recorded at three different depths (*z*-axis positions).

### 3.12. Animal Models

Animal studies were conducted using an approved protocol in accordance with the French ethics committee of Champagne-Ardenne and the French research ministry (APAFIS# 4373_v1). Female athymic BALB/c nu/nu mice were obtained from Charles River Laboratories (Ecully, France) at six weeks of age. Breast cancer MDA-MB-231 was orthotopically injected (5 × 10^6^ cells) into the fourth mammary abdominal gland. Mice followed a special diet with an alfalfa-free diet (Envigo, Gannat, France) to reduce auto-fluorescence. When tumors reached 200 mm^3^, mice were imaged with FMT 4000 (PerkinElmer, Villebon-sur-Yvette, France).

### 3.13. In Vivo and Ex Vivo Fluorescence Imaging

For in vivo and ex vivo fluorescence experiments, 100 µL of Au@DTDTPA-Cy5 were injected into the tail vein of anesthetized mice. Mice were imaged in the decubitus lateral position with an FMT 4000 (PerkinElmer, Villebon-sur-Yvette, France) small animal scanner using a 635 nm excitation wavelength and a 650–670 emission filter. Then, 3D trans-illumination acquisitions were performed 30 min post-injection. Images were captured and reconstructed using TrueQuant (v3.1) software (PerkinElmer, Waltham, MA, USA).

## 4. Conclusions

The presence of DTDTPA at the surface of gold nanoparticles plays a crucial role in the growth control of the metallic core during the reduction of the gold salt by NaBH_4_, in the immobilization of metal ions used for medical imaging (MRI and nuclear imaging), and in the colloidal stability of the nanoparticles. This work demonstrates that DTDTPA ligands also act as grafting sites for post-functionalization with aminated NIR dye owing to the COOH moieties (3 COOH per ligand). The grafting of NIR Cy5-NH_2_ dye onto the DTDTPA organic shell confers efficient fluorescence properties to the nanoparticles. This new development in nanoparticle design offers the possibility to monitor internalization and localization in cells by fluorescence microscopy, as well as biodistribution in small animals using fluorescence imaging. Although the internalization of non-labeled Au@DTDTPA nanoparticles can be monitored by HAADF-STEM, the follow-up by fluorescence imaging appears more attractive for at least two main reasons: (i) the fluorescence imaging is characterized by its ease of implementation and (ii) in contrast to HAADF-STEM, fluorescence imaging can be efficiently exploited for in vivo study in real time.

The surface modification of Au@DTDTPA nanoparticles by NIR dyes exerts no (or only a little) influence on their behavior in the presence of cells. The uptake (amount of gold nanoparticles in cells) and internalization kinetics are almost the same for HeLa and U87 MG cells than those observed for non-functionalized Au@DTDTPA nanoparticles. However, the fluorescence properties of Au@DTDTPA-Cy5 nanoparticles confer a crucial advantage for monitoring their fate after internalization in cells. The experiments performed with MitoTracker Green reveal the presence of gold nanoparticles in the vicinity of mitochondria. Knowing where the nanoparticles are constitutes a real advantage for exploiting the radiosensitizing effect of gold nanoparticles. In a previous study, we demonstrated that the most crucial factor for an efficient control of tumor growth is the localization of the radiosensitizers (gadolinium-based nanoparticles) rather than their concentration [18].

The post-functionalization of Au@DTDTPA nanoparticles by NIR dyes exerts also no influence on the biodistribution of the nanoparticles since the behavior after intravenous injection into tumor-bearing mice is the same for Au@DTDTPA-Cy5 and Au@DTDTPA [27,28]. The preferential accumulation in the tumor and the renal clearance are preserved after the post-functionalization. Although they rest on paradoxical phenomenon, both characteristics (tumor accumulation and renal clearance) are essential for therapeutic applications and, in peculiar, for radiosensitization [23]. The renal clearance allows the removal of non-biodegradable radiosensitizer excess. The radiosensitization will therefore be restricted to the tumor for better selectivity of the radiotherapy treatment.

In summary, the post-functionalization of Au@DTDTPA with NIR organic fluorophores (aminated Cy-5) opens the perspective of investigating the relationship between sub-cellular localization, in vivo biodistribution, and improvement of X-ray performances using these gold nanoparticles, a key step in the design of more efficient nanotheranostic agents.

## Abbreviations

Au@DTDTPA	gold nanoparticles coated by linear chelator
Au@DTDTPA-Cy5	gold nanoparticles coated by linear chelator and functionalized with cyanine 5
DTPA	diethylenetriaminepentaacetic acid (linear chelator)
DTDTPA	dithiolated derivative of diethylenetriaminepentaacetic acid
Au@TADOTAGA	gold nanoparticles coated by macrocyclic chelator
DOTA	1,4,7,10-tetraazacyclododecane-1,4,7,10-tetraacetic acid
DOTAGA	1,4,7,10-tetraazacyclododecan-1-glutaric acid-4,7,10-triacetic acid
TADOTAGA	DOTAGA functionalized by thioctic acid
Cy5-NH_2_	aminated derivative of cyanine 5
Z	atomic number
vs.	versus
MRI	magnetic resonance imaging
SPECT	single-photon emission computed-tomography
MRT	microbeam radiation therapy
pi	post-injection
EDC	*N*-(3-Dimethylaminopropyl)-*N*′-ethylcarbodiimide
NHS	*N*-hydroxysuccinimide
MWCO	molecular weight cut-off
TEM	transmission electron microscopy
UV	ultraviolet
ICP-OES	inductively coupled plasma-optical emission spectrometry

## References

[1] Hainfeld J.F., Slatkin D.N., Smilowitz H.M. (2004). The use of gold nanoparticles to enhance radiotherapy in mice. Phys. Med. Biol..

[2] Hainfeld J.F., Smilowitz H.M., O’Connor M.J., Dilmanian F.A., Slatkin D.N. (2013). Gold nanoparticle imaging and radiotherapy of brain tumors in mice. Nanomedecine.

[3] Schuemann J., Berbeco R., Chithrani D.B., Cho S.H., Kumar R., McMahon S.J., Sridhar S., Krishnan S. (2016). Roadmap to clinical use of gold nanoparticles for radiation sensitization. Int. J. Radiat. Oncol. Biol. Phys..

[4] Haume K., Rosa S., Grellet S., Śmiałek M.A., Butterworth K.T., Solov’yov A.V., Prise K.M., Golding J., Mason N.J. (2016). Gold nanoparticles for cancer radiotherapy: A review. Cancer Nano.

[5] Rosa S., Connolly C., Schettino G., Butterworth K.T., Prise K.M. (2017). Biological mechanisms of gold nanoparticle radiosensitization. Cancer Nano.

[6] Kuncic Z., Lacombe S. (2018). Nanoparticle radio-enhancement: Principles, progress and application to cancer treatment. Phys. Med. Biol..

[7] McMahon S.J., Hyland W.B., Muir M.F., Coulter J.A., Jain S., Butterworth K.T., Schettino G., Dickson G.R., Hounsell A.R., O’Sullivan J.M. (2011). Biological consequences of nanoscale energy deposition near irradiated heavy atom nanoparticles. Sci. Rep..

[8] Kobayashi K., Usami N., Porcel E., Lacombe S., Le Sech C. (2010). Enhancement of radiation effect by heavy elements. Mutat. Res..

[9] Butterworth K.T., McMahon S.J., Currell F.J., Prise K.M. (2012). Physical basis and biological mechanisms of gold nanoparticle radiosensitization. Nanoscale.

[10] Retif P., Pinel S., Toussaint M., Frochot C., Chouikrat R., Bastogne T., Barberi-Heyob M. (2015). Nanoparticles for radiation therapy enhancement: The key parameters. Theranostics.

[11] Liu Y., Zhang P., Li F., Jin X., Li J., Chen W., Li Q. (2018). Metal-based nanoenhancers for future radiotherapy: Radiosensitizing and synergistic effects on tumor cells. Theranostics.

[12] Scheinberg D.A., Villa C.H., Escorcia F.E., McDevitt M.R. (2010). Conscripts of the infinite armada: Systemic cancer therapy using nanomaterials. Nat. Rev. Clin. Oncol..

[13] Min Y., Caster J.M., Eblan M.J., Wang A.Z. (2015). Clinical translation of nanomedicine. Chem. Rev..

[14] Kunjachan S., Ehling J., Storm G., Kiessling F., Lammers T. (2015). Noninvasive imaging of nanomedicines and nanotheranostics: Principles, progress, and prospects. Chem. Rev..

[15] Dou Y., Guo Y., Li X., Li X., Wang S., Wang L., Lv G., Zhang X., Wang H., Gong X. (2016). Size-tuning ionization to optimize gold nanoparticles for simultaneous enhanced CT imaging and radiotherapy. ACS Nano.

[16] Porcel E., Liehn S., Remita H., Usami N., Kobayashi K., Furusawa Y., Le Sech C., Lacombe S. (2010). Platinum nanoparticles: A promising material for future cancer therapy?. Nanotechnology.

[17] Le Duc G., Miladi I., Alric C., Mowat P., Bräuer-Krisch E., Bouchet A., Khalil E., Billotey C., Janier M., Lux F. (2011). Toward an image-guided microbeam radiation therapy using gadolinium-based nanoparticles. ACS Nano.

[18] Dufort S., Le Duc G., Salomé M., Bentivegna V., Sancey L., Bräuer-Krisch E., Requardt H., Lux F., Coll J.-L., Perriat P. (2016). The high radiosensitizing efficiency of a trace of gadolinium-based nanoparticles in tumors. Sci. Rep..

[19] Lux F., Mignot A., Mowat P., Louis C., Dufort S., Bernhard C., Denat F., Boschetti F., Brunet C., Antoine R. (2011). Ultrasmall rigid particles as multimodal probes for medical applications. Angew. Chem. Int. Ed..

[20] Alqathanni M., Blencowe A., Geso M., Ibbott G. (2016). Quantitative 3D Determination of Radiosensitization by Bismuth-Based Nanoparticles. J. Biomed. Nanotechnol..

[21] Detappe A., Thomas E., Tibbitt M.W., Kunjachan S., Zavidij O., Parnandi N., Reznichenko E., Lux F., Tillement O., Berbeco R. (2017). Ultrasmall silica-based bismuth gadolinium nanoparticles for dual magnetic resonance–computed tomography image guided radiation therapy. Nano Lett..

[22] Soo Choi H., Liu W., Misra P., Tanaka E., Zimmer J.P., Itty Ipe B., Bawendi M.G., Frangioni J.V. (2007). Renal clearance of quantum dots. Nat. Biotechnol..

[23] Yu M., Zheng J. (2015). Clearance pathways and tumor targeting of imaging nanoparticles. ACS Nano.

[24] Debouttière P.-J., Roux S., Vocanson F., Billotey C., Beuf O., Favre-Réguillon A., Lin Y., Pellet-Rostaing S., Lamartine R., Perriat P. (2006). Design of gold nanoparticles for magnetic resonance imaging. Adv. Funct. Mater..

[25] Alric C., Taleb J., Le Duc G., Mandon C., Billotey C., Le Meur-Herland A., Brochard T., Vocanson F., Janier M., Perriat P. (2008). Gadolinium chelate coated gold nanoparticles as contrast agents for both X-ray computed tomography and magnetic resonance imaging. J. Am. Chem. Soc..

[26] Arifin D.R., Long C.M., Gilad A.A., Alric C., Roux S., Tillement O., Link T.W., Arepally A., Bulte J.W.M. (2011). Trimodal gadolinium-gold microcapsules containing pancreatic islet cells restore normoglycemia in diabetic mice and can be tracked by using US, CT, and positive-contrast MR imaging. Radiology.

[27] Alric C., Miladi I., Kryza D., Taleb J., Lux F., Bazzi R., Billotey C., Janier M., Perriat P., Roux S. (2013). The biodistribution of gold nanoparticles designed for renal clearance. Nanoscale.

[28] Miladi I., Alric C., Dufort S., Mowat P., Dutour A., Mandon C., Laurent G., Bräuer-Krisch E., Herath N., Coll J.-L. (2014). The in vivo radiosensitizing effect of gold nanoparticles based MRI contrast agents. Small.

[29] Laurent G., Bernhard C., Dufort S., Jiménez Sánchez G., Bazzi R., Boschetti F., Moreau M., Vu T.H., Collin B., Oudot A. (2016). Minor changes in the macrocyclic ligands but major consequences on the efficiency of gold nanoparticles designed for radiosensitization. Nanoscale.

[30] Butterworth K.T., Nicol J.R., Ghita M., Rosa S., Chaudhary P., McGarry C.K., McCarthy H.O., Jiménez Sánchez G., Bazzi R., Roux S. (2016). Preclinical evaluation of gold-DTDTPA nanoparticles as theranostic agents in prostate cancer radiotherapy. Nanomedicine.

[31] Caravan P., Ellison J.J., McMurry T.J., Lauffer R.B. (1999). Gadolinium(III) chelates as MRI contrast agents:  structure, dynamics, and applications. Chem. Rev..

[32] Miyawaki A. (2003). Visualization of the spatial and temporal dynamics of intracellular signaling. Dev. Cell.

[33] Hermanson G.T. (2008). Bioconjugate Techniques.

[34] Ghosh S.K., Pal T. (2007). Interparticle coupling effect on the surface plasmon resonance of gold nanoparticles:  from theory to applications. Chem. Rev..

[35] Chen S., Kimura K. (1999). Synthesis and characterization of carboxylate-modified gold nanoparticle powders dispersible in water. Langmuir.

[36] Wu Z., Jin R. (2010). On the Ligand’s Role in the Fluorescence of Gold Nanoclusters. Nano Lett..

[37] Duff D.G., Baiker A., Edwards P.P. (1993). A new hydrosol of gold clusters. J. Chem. Soc. Chem. Commun..

[38] Faure A.-C., Dufort S., Josserand V., Perriat P., Coll J.-L., Roux S., Tillement O. (2009). Control of the in vivo Biodistribution of Hybrid Nanoparticles with Different Poly(ethylene glycol) Coatings. Small.

[39] Kobayashi H., Ogawa M., Alford R., Choyke P.L., Urano Y. (2010). New strategies for fluorescent probe design in medical diagnostic imaging. Chem. Rev..

[40] Brust M., Fink J., Bethell D., Schiffrin D.J., Kiely C. (1995). Synthesis and reactions of functionalised gold nanoparticles. J. Chem. Soc. Chem. Commun..

